# Protection of Astaxanthin in Astaxanthin Nanodispersions Using Additional Antioxidants

**DOI:** 10.3390/molecules18077699

**Published:** 2013-07-02

**Authors:** Navideh Anarjan, Imededdine Arbi Nehdi, Chin Ping Tan

**Affiliations:** 1 Department of Engineering, East Azarbaijan Science and Research Branch, Islamic Azad University, Tabriz 1547, Iran; E-Mail: anarjan@iauasrb.ac.ir; 2 Chemistry Department, College of Science, King Saud University, Riyadh 1145, Saudi Arabia; E-Mail: inahdi@ksu.edu.sa; 3 Department of Food Technology, Faculty of Food Science and Technology, Universiti Putra Malaysia, 43400 UPM Serdang, Selangor, Malaysia

**Keywords:** astaxanthin nanodispersions, chemical stability, ascorbic acid, α-tocopherol

## Abstract

The protective effects of α-tocopherol and ascorbic acid on astaxanthin in astaxanthin nanodispersions produced via a solvent-diffusion technique and stabilized by a three-component stabilizer system, were studied either individually or in combination by using response surface methodology. Generally, both α-tocopherol and ascorbic acid could retard the astaxanthin degradation in astaxanthin nanodispersions. The results showed that the using α-tocopherol and ascorbic acid can be more efficient in increasing the chemical stability of nanodispersions in comparison to using them individually. Using a response surface methodology (RSM) response optimizer, it was seen that addition of ascorbic acid (ascorbic acid/astaxanthin w/w) and α-tocopherol (α-tocopherol/astaxanthin w/w) in proportions of 0.4 and 0.6, respectively, would give the maximum chemical stability to the studied astaxanthin nanodispersions.

## 1. Introduction

Carotenoids are the most important pigments in Nature that are responsible for the various colours of different photosynthetic organisms [1,2,3]. Because of carotenoids’ general inability to be synthesised in animal cells, they must be obtained from the diet [1,4]. Recent studies have considered carotenoids with no pro-vitamin A activity (such as astaxanthin) because of their importance to cell survival, growth, reproduction, pigmentation and protection against light sensitisation [5]. Astaxanthin, which can be found in different aquatic animals (and, therefore, seafoods), including lobster, salmon, red sea bream, trout, shrimp, and fish eggs, has superior antioxidant activity due to existence of both hydroxyl and ketonic end groups on each ionone ring of its chemical structure [2]. It can react with a scavenger radical either by removing one of the latter’s electrons or by adding itself to another molecule to pair its own unpaired electron, thereby forming an adduct [6].

However, like other carotenoids, the low water solubility of this functional lipid compound has made its use in water based food formulations problematic. Furthermore, these compounds may be prone to reduced bioavailability [7]. Therefore, it is important to find solutions to this problem. One of the most important applications of nanotechnology in the food and nutrition industries is in the design and development of novel functional food ingredients with improved water solubility, thermal stability, oral bioavailability, sensory attributes and physiological performances. Nanodispersion systems are a few of the nanoscale systems currently being investigated for commercial applications in this regard [3,8].

It is noteworthy that like other carotenoids, astaxanthin is unstable because of its susceptibility to light, oxygen, and autooxidation [9,10]. Generally the combination of different antioxidants may act additively or even synergistically. The synergistic antioxidant activities of carotenoids with α-tocopherol and ascorbic acid have already been confirmed by various previous researches [10,11,12]. It was shown that α-tocopherol, that is an important natural antioxidant in living cells and the most important lipid-soluble radical scavenger in membranes and plasma, can protect carotenoids from autooxidation [10,13,14]. Although water-soluble ascorbic acid was less reactive with lipid radicals than hydrophobic astaxanthin, it was able to interact with the oxidized forms of astaxanthin to regenerate astaxanthin in a time-course reaction [15].

Therefore, in this research astaxanthin was first incorporated in nanodispersion system to produce water dispersible astaxanthin nanoparticles through a solvent diffusion technique. A mixture of a small molecular emulsifier (Polysorbate 20), protein (sodium caseinate) and polysaccharide (gum Arabic) with optimized proportions was used as a three-component stabilizer system in production of astaxanthin nanoparticles [16]. Then the effects of additional α-tocopherol and ascorbic acid on astaxanthin stability of produced nanodispersions were investigated and at last, the concentrations of these additional antioxidant compounds were optimized using response surface methodology.

## 2. Results and Discussion

**Figure 1:**
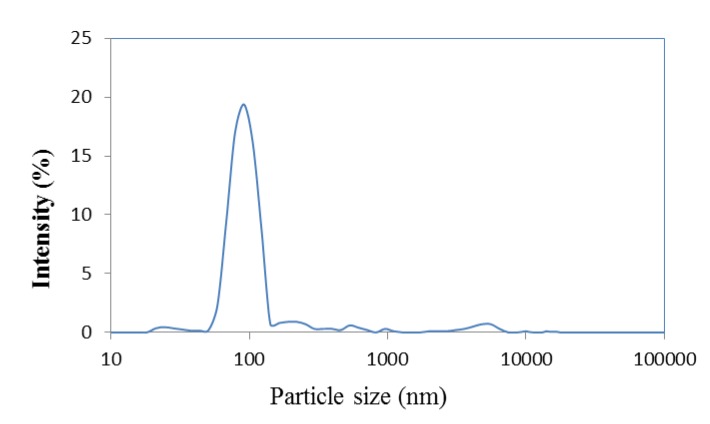
Particle size distribution of freshly produced astaxanthin nanodispersion.

Astaxanthin nanodispersions were produced using emulsification solvent-diffusion method. For this method, the first step of particle preparation is organic phase dispersion of globules in aqueous phase at high stirring speed. Once the emulsion is formed, the submicron droplets are then diluted in water by removing the organic phase, and the interaction between the emulsion droplets and the dilution phase is referred to as a modification of phase equilibrium and solvent diffusion, which leads to precipitation of astaxanthin and production of astaxanthin particles. Since the particle size of produced astaxanthin nanodispersions (measured using a Zetasizer Nano ZS, Malvern Instruments Ltd., Worcestershire, UK), was in nanometre ranges (98.3 ± 4.27 nm), it can be concluded that the high shear stress due to the emulsification step have guaranteed the submicron droplet formation of astaxanthin in studied dispersion system. The particle size distribution of freshly produced astaxanthin nanodispersions was shown in Figure 1.

The astaxanthin degradation rates in all studied samples can be fit by first-order kinetic equations with coefficients of determination (*R^2^*) higher than 0.8 (Table 1). Therefore, the degradation rate of astaxanthin (r) in nanodispersions can be written as:

(1) $$\text{r}=\frac{dC}{dt}=kC\to -\frac{dC}{C}=k\;t\to ln\;\frac{C}{C_{0}}-=-\;kt$$

where C is the concentration of astaxanthin at time t, C_0_ is initial concentration of astaxanthin (100 mg/L) and k is the rate constant.

In autooxidation of astaxanthin through a free radical chain mechanism, the astaxanthin radical is formed by an attack of radicals, in the initiation step, then the carbon-centered, carotenoid radical reacts with oxygen to give astaxanthin peroxyl radical, which attacks another astaxanthin molecule and abstracts a hydrogen atom to give astaxanthin hydroperoxide and at the same time a new astaxanthin radical starts the propagation sequence over again. Consequently, many molecules of astaxanthin can be oxidized to astaxanthin hydroperoxides for every initiation event.

The propagation cycle can be broken by termination reactions, which result in the destruction of free radicals. The autooxidation of astaxanthin can be suppressed by inhibiting the initiation step or accelerating the termination step, which can be provided by addition of antioxidants that are divided into preventive antioxidants and chain-breaking antioxidants categories. Whereas preventive antioxidants neutralize the active species and possible precursors of free radicals and thereby suppress the generation of free radicals and reduce the rate of chain initiation, the chain-breaking antioxidants can scavenge oxygen-centred radicals and suppress the autooxidation [17,18].

**Table 1:** Rate constant and coefficient of determinations for astaxanthin degradation of optimum astaxanthin nanodispersions diluted in deionized water with different concentrations of additional antioxidant compounds (followed a first order kinetic equation).

Antioxidant		Antioxidant concentrations (mg/L)				
		0	5	10	50	100
α-tocopherol	Rate constant (week^−1^)	0.3685 ^aA^	0.0926 ^bB^	0.0140 ^cB^	0.0190 ^dB^	0.0125 ^eB^
	SE Coef. (n = 3) ^1^	0.0042	0.0065	0.0010	0.0000	0.0008
	*R^2^*	0.9915	0.9857	0.8977	0.9526	0.8139
Ascorbic acid	Rate constant (week^−1^)	0.3685 ^aA^	0.2528 ^cA^	0.2106 ^dA^	0.2028 ^eA^	0.2854 ^bA^
	SE Coef. (n = 3) ^1^	0.0551	0.0004	0.0043	0.0029	0.0000
	*R^2^*	0.9915	0.9506	0.9822	0.9793	0.9856

^1^ SE Coef.: Standard error of coefficient (rate constant), n: number of replications; ^a–e^ Different letters show significant differences (*p* < 0.05) among response values in which the comparison tests were performed between similar responses of each column (different antioxidant concentrations). ^A–B^ Different letters show significant differences (*p* < 0.05) between response values in which the comparison test was performed between similar responses of each row (different antioxidant types).

Ascorbic acid and α-tocopherol are two common antioxidant molecules used in different food and beverage formulations. The effect of water-soluble ascorbic acid as an antioxidant is complicated because of its multiple functions. Ascorbic acid is oxidized by oxygen to give the ascorbate radical anion and hydrogen peroxide, although the precise molecular mechanism remains controversial. Ascorbic acid also can react with iron and reduce ferric ion to ferrous ion which lead to decomposition of hydroperoxide and hydrogen peroxide to give alkoxyl and hydroxyl radicals respectively. Accordingly, ascorbic acid may function as a prooxidant in the presence of iron and hydrogen peroxide or hydroperoxide. On the other hand, α-tocopherol is a fat-soluble phenolic compound that can scavenge the chain-carrying peroxyl radicals rapidly and interrupts the chain propagation [19]. The high activity of α-tocopherol as an antioxidant stems from the high reactivity of α-tocopherol toward oxygen-centred radicals. In fact, α-tocopherol has much higher rate constant than other synthetic phenolic antioxidants [18,20].

Table 1 shows the astaxanthin loss rate in nanodispersions with deionized water containing α-tocopherol and acid ascorbic in different concentrations. The astaxanthin degradation rate in all studied diluted nanodispersion systems in this part were also followed first order kinetic equations. As can be seen in Table 1, the addition of α-tocopherol was more efficient than ascorbic acid in prevention of astaxanthin degradation of diluted optimum formulated nanodispersions. Therefore, as also reported in previous researchers, the presence of lipophilic antioxidants in carotenoid nanodispersion systems can inhibit carotenoid oxidation more effectively than hydrophilic antioxidants [21]. They can react with free radical intermediates of carotenoid peroxidation and with the peroxides and consequently make the carotenoids inactive against these active molecules. They can also act as a reducing agent for transition metals. The major biochemical function of α-tocopherol in these systems is its reactivity with possible presented organic peroxyl radicals in environment. Additionally, tocopherols are the most effective antioxidant at high oxygen tensions [21,22]. Additionally, Table 1 illustrated that in the systems containing antioxidants, astaxanthin degradation could also be well explained by first-order kinetics (high *R^2^* values). Increasing the concentrations of added antioxidants up to certain concentrations could caused a decrease in astaxanthin degradation of systems, but further increase in their concentrations did not affect the astaxanthin loss rates considerably or affect it inversely. Due to synergistic antioxidant effect of ascorbic acid and α-tocopherol, which was reported in previous reports [23,24,25], a CCD was conducted to study the effects of their combinational effects on retarding of astaxanthin loss of diluted optimum formulated nanodispersions in deionized water.

Response-surface analysis provided empirically significant (*p* < 0.05) models for estimating the rate constant of astaxanthin degradation during the storage at 5 °C as function of the ascorbic acid and α-tocopherol concentrations in the prepared nanodispersions. As a result, the optimization process predicted an optimum level for these independent variables that resulted in the desirable goals. The interaction term were dropped because of its insignificant effect on studied response value (*p* > 0.05). However, the linear term of ascorbic acid concentration were included in the final reduced despite of its insignificancy (*p* > 0.05), because its quadratic terms were found to be significant (*p* < 0.05).

Table 2 contains the corresponding *R^2^*, adjusted *R^2^* values of the regression equations and significance of each term in final reduced model which were determined using the F-ratio and p-values. The relationship between astaxanthin degradation rate constant (week^−1^) and concentrations of ascorbic acid and α-tocopherol could be explained significantly (*p* < 0.05) by a second-order polynomial regression model. The main and quadratic effect of α-tocopherol concentration had a highest significant effect (highest F-ratio) on studied response than of ascorbic acid. Therefore, α-tocopherol concentration was considered as critical parameter to control astaxanthin degradation of nanodispersions with additional antioxidants, diluted in deionized water during storage. The final reduced model fitted to the experimental data, was a valid statistical empirical model only in the selected ranges [26].

**Table 2:** Regression coefficients, R^2^, adjusted R^2^ and probability values for the final reduced models.

Regression coefficients	Coefficients	F-ratio	P-value
β_0_	20.6923	44.42	0.000^b^
β_1_	−0.1490	2.84	0.138
β_2_	−0.6854	57.35	0.000^b^
β_11_	0.0028	10.89	0.011^b^
β_22_	0.0059	47.83	0.000^b^
β_12_	-	-	-
R^2^	91.5%	21.64 (Regression)	0.000 (Regression)
R^2^-adj	87.3%		

β_0_ is a constant, β_i_, β_ii_ and β_ij_ are the linear, quadratic and interaction coefficients of the quadratic polynomial equation, respectively. 1: Concentration of ascorbic acid 2: Concentration of α-tocopherol; ^b^ Significant (*p* < 0.05).

Generally, the results demonstrated that the addition of antioxidants, either individually or in combination, could decrease the astaxanthin degradation rate constant in all prepared astaxanthin nanodispersions. The astaxanthin degradation rate constant in all samples were less than astaxanthin degradation rate constant of optimum formulated nanodispersion without addition of any antioxidant agents. The astaxanthin degradation rate constant was negatively associated with the linear effects of α-tocopherol, and positively associated with the quadratic effects of both ascorbic acid and α-tocopherol concentrations.

**Figure 2:**
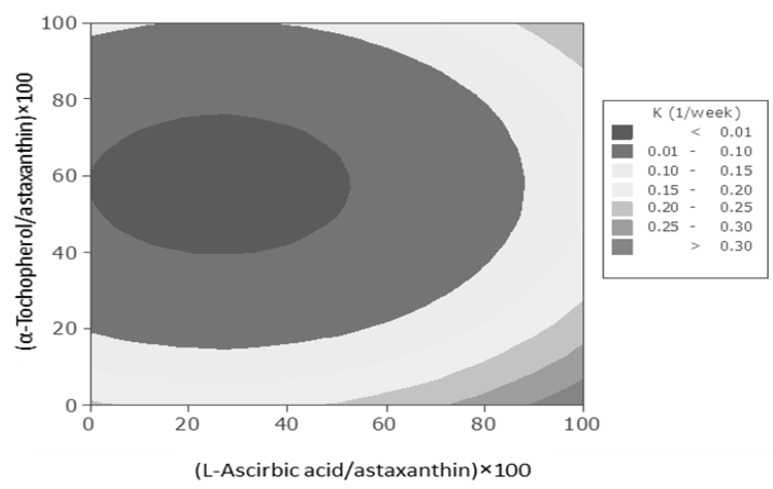
.Contour plot for astaxanthin degradation rate constant (week^−1^) in astaxanthin nanodispersions as a function of added ascorbic acid and α-tocopherol concentrations.

As shown in Figure 2, the combination of the highest levels of ascorbic acid and the least levels of α-tocopherol or *vice versa* led to highest astaxanthin degradation rates in these systems. In contrast, the considerable decrease in astaxanthin degradation rate took place due to combination of middle levels of α-tocopherol concentration with low levels of ascorbic acid concentrations. Despite of relatively low antioxidant activity of ascorbic acid when it was added individually to diluted astaxanthin nanodispersions, it could drastically enhance the antioxidant activity when it was combined with α-tocopherol. In combination of low concentrations of ascorbic acid with relatively middle concentrations of α-tocopherol, the ascorbic acid might reduce the tocopheroxyl radical formed from tocopherol during the scavenging of free radicals *in vivo*, which permits a single molecule of α-tocopherol to scavenge many radicals and also links ascorbic acid to the protection of astaxanthin against free-radical damage. It was also reported that in these systems, both α-tocopherol and ascorbic acid can scavenge the peroxyl radicals, but α-tocopherol scavenges the radicals faster than ascorbic acid. However, the α-tocopherol radical formed is reduced by ascorbic acid to regenerate α-tocopherol. Thus, α-tocopherol is not consumed at first and only ascorbic acid disappears [18].

Astaxanthin might scavenge the existed radicals in the system and produce radicals which may undergo following competing reactions; it may react with another peroxyl radical to give a stable adduct, react with another astaxanthin to give a dimmer or react with α-tocopherol or ascorbic acid to regenerate astaxanthin and produce α-tocopherol or ascorbic acid radicals. The reduction of astaxanthin with α-tocopherol was more preferable than ascorbic acid. The same procedure would be happened to α-tocopherol radicals. The mentioned path reactions are determined by the reactivities, concentrations and accessibilities components. It could be suggested that in studied binary mixture of ascorbic acid and α-tocopherol, high concentrations of ascorbic acid would interrupt the reduction of astaxanthin molecules by α-tocopherol in regeneration process of astaxanthin. Therefore, the synergistic effect of ascorbic acid and α-tocopherol in studied system were found to be dependant of their concentrations [18,24,27,28].

The results also showed that high concentrations of ascorbic acid (higher than 60 mg/L), led to physical instability in diluted nanodispersions due to decreasing in pH of system that approached to isoelectric pH of used stabilizer system. The regression equations showed that, at the lower levels of studied factors, increasing the concentrations of both antioxidants caused a decrease in astaxanthin autooxidation rate constant, but further increasing in concentrations affected the astaxanthin degradation inversely and led to increase in astaxanthin loss of diluted nanodispersions. Therefore, the optimum values for antioxidant concentrations to produce diluted astaxanthin nanodispersions with minimum autooxidation of astaxanthin and maximum chemical stability, fell between the low concentrations for ascorbic acid and middle concentrations for α-tocopherol. The same results had also been obtained in previous researchers which were done on synergic effect of different groups of water soluble and fat soluble antioxidants [24,27].

In this study, concentrations of antioxidants in optimum astaxanthin nanodispersions that led to considerable chemical stability were considered as most favourable, if they led to a considerable increase in chemical stability of system without any changes in their physical stability. A numerical optimization was also carried out to check the exact optimum levels of the independent variables leading to the desired response goal. It showed that the diluted astaxanthin nanodispersion with the highest chemical stability was predicted to be obtained by using 40 mg/L ascorbic acid and 60 mg/L α-tocopherol as additives during the preparation of astaxanthin nanodispersions. Under the optimum conditions, the corresponding predicted response value for astaxanthin degradation rate constant was found to be less than 0.00001 (week^−1^). No significant (*p* > 0.05) difference that were observed between the initial and after storage (four weeks at 5 °C) astaxanthin concentrations of nanodispersions containing optimized antioxidants additives, confirmed the high chemical stability of produced astaxanthin nanodispersions. The experimental and predicted values of astaxanthin loss rate constants of diluted optimum nanodispersions in deionized water were shown in Table 3.

**Table 3:** Experimental and predicted values of astaxanthin loss rate of diluted optimum nanodispersions in deionized water obtained from the applied CCD for optimization the concentrations of additional antioxidants.

Treatment runs	Y _exp_	Y _pre_	Y _exp_- Y _pre_
1	0.0092	0.0093	−0.0001
2	0.0095	0.0093	0.0002
3	0.1143	0.1136	0.0007
4	0.2028	0.2033	−0.0005
5	0.0091	0.0093	−0.0002
6	0.0937	0.0945	−0.0008
7	0.0093	0.0093	0.0000
8	0.0075	0.0073	0.0002
9	0.1550	0.1524	0.0026
10	0.0190	0.0188	0.0002
11	0.0092	0.0093	−0.0001
12	0.1834	0.1875	−0.0041
13	0.1765	0.1888	−0.0123

Y_exp:_experimental data, Y_pre:_ predicted data, Y _exp_- Y _pre_: residual.

No significant difference (*p* > 0.05) between experimental and predicted value of astaxanthin degradation rate constants confirmed the sufficiency of the corresponding regression model used for predicting the variation of studied response.

## 3. Experimental

### 3.1. Materials

Synthetic *trans*-astaxanthin (>85%) was purchased from Kailu Ever Brilliance Biotechnology Co., Ltd. (Beijing, China). Polyoxyethylene sorbitan monolaurate (Polysorbate 20), phosphate buffer (pH=7), sodium caseinate (SC), sodium azide, analytical and HPLC-grade dichloromethane, acetone, methanol and acetonitrile were purchased from Fisher Scientific (Leicestershire, UK). Gum Arabic (GA) was donated by Merck Co. (Darmstadt, Germany). α-Tocopherol (95%) and L-ascorbic acid (reagent grade) were acquired from Sigma (St. Louis, MO, USA).

### 3.2. Preparation of Astaxanthin Nanodispersions

0.02% w/w sodium azide and 2.5% w/w of stabilizer (composed 29% w/w Polysorbate 20, 65% w/w sodium caseinate and 6% w/w gum Arabic) were dissolved at 40 °C in 0.05 M phosphate buffer (pH 7) under magnetic stirring for overnight and then centrifuged for 5 min at 800 × *g* using a KOBOTA 2010 (Tokyo, Japan) centrifuge. The organic phase that composed of dissolved astaxanthin (0.08% w/w) in mixture of 38% w/w dichloromethane and 62% w/w acetone was added to the aqueous phase at the organic: aqueous phase ratio of 11.5% w/w, and homogenized in a conventional homogenizer (Silverson, L4R, Buckinghamshire, UK) at 5,000 rpm for 5 min. The resulting coarse emulsion was then passed three times through a high-pressure homogenizer (APV, Crawley, UK) at 30 MPa to prepare a fine nanoemulsion [9]. According to Table 1 and Table 4, ascorbic acid and/or α-tocopherol (0–100 mg/L) were also dissolved in some samples. 

**Table 4:** Central composite design (CCD) matrix for optimisation the antioxidant components.

Treatment runs	α-tocopherol/astaxanthin (w/w)	Ascorbic acid/astaxanthin (w/w)
1(C)	0.50	0.50
2(C)	0.50	0.50
3	0.15	0.15
4	0.50	0.00
5(C)	0.50	0.50
6	0.85	0.85
7(C)	0.50	0.50
8	0.15	0.85
9	0.50	1.00
10	0.00	0.50
11(C)	0.50	0.50
12	1.00	0.50
13	0.85	0.15

(C) Centre point.

The conversion of nanoemulsion to nanodispersion was performed by removing the solvents from the systm using a rotary evaporation (Eyela NE-1001, Tokya Rikakikai Co. Ltd., Tokyo, Japan) at 150 Pa and 25 °C and 100 rpm. The final concentration of astaxanthin in prepared nanodispersion was set at 100 mg/L by dilution the system using deionized water. For each analysis, glasses were filled and tightly sealed and placed in the dark at 5 °C for the 4 weeks of storage and the astaxanthin concentration was quantified periodically (every 2 days).

### 3.3. Determination of Astaxanthin Content

For sample preparation, in an amber vial with a screw top, 0.5 mL of sample was dissolved in 2 mL of a mixture of methanol and dichloromethane (50:50 v/v) and agitated for 15 min. The mixture was centrifuged for 5 min at 800 × *g*, using a Kobota 2010 (Tokyo, Japan) centrifuge at room temperature, and the extract was decanted. The extraction procedure was carried out two more times. Methanol was then added to extract to bring up the samples to 10 mL. A sample aliquot was filtered with a membrane filter, and 40 μL of filtered sample aliquot with membrane filter, was injected into the HPLC.

An Agilent liquid chromatography system (Agilent Technologies 1200 Series, Waldbroon, Germany), equipped with a G13150 Diode Array Detector and a Nova-Pak^®^ C18 (3.9 × 300 mm) Waters HPLC column was employed for HPLC analysis, using an isocratic mobile phase consisting of 85% v/v methanol, 5% v/v 5% v/v dichloromethane, acetonitrile and 5% v/v water. The detection was performed at 480 nm [29]. The calibration of peak area versus astaxanthin concentration was linear in the range of measured concentrations (*R^2^* = 0.9888). The measurement was reported as an average of three individual injections.

### 3.4. Statistical Analysis

For investigation of the effects of antioxidants on astaxanthin stability, linear regression analysis was used to find the degradation rate constants for astaxanthin in all studied conditions, using the Minitab v. 14 Statistical Package (Minitab Inc., State College, PA, USA). Subsequently, RSM was applied to evaluate the effects of the α-tocopherol (x_1_) and ascorbic acid (x_2_) concentrations on astaxanthin stability in produced nanodispersions. The corresponding values for the two factors varying at three levels (2^3^, α = 1.414) and their combinations for each test sample, determined using a central composite design (CCD), are displayed in Table 4. Because the degradation rate of astaxanthin followed first-order kinetics, the rate constant for astaxanthin degradation in samples was considered as response variable [1]. The advantage of present CCD was to concurrently study the main and interaction effects of two independent variables on the response variable studied. The CCD includes an imbedded factorial design with center points that is augmented with a group of “star points” that allows estimation of curvature [30]. The model terms were selected or rejected based on the p-value at a 95% confidence level. The optimum condition (within the experimental range) for minimal astaxanthin loss was obtained using the response optimiser function in MINITAB 14.

## 4. Conclusions

In this study, astaxanthin nanodispersions were prepared using solvent-diffusion technique. The effects of addition of α-tocopherol and ascorbic acid in different concentrations on decreasing the rate of astaxanthin lost in prepared nanodispersions were studied. The results showed that however, α-tocopherol is more proficient in retarding astaxanthin decomposition compared to ascorbic acid, and there is an optimum concentrations for either α-tocopherol or ascorbic acid in reducing the astaxanthin degradation rates in nanodispersions. The single, quadratic and interaction effects of additional antioxidant concentrations’ on degradation rates of astaxanthin in produced nanodispersion systems were evaluated using RSM. The optimization procedure reported the 40 mg/L ascorbic acid (or ascorbic acid/astaxanthin: 0.4) and 60 mg/L α-tocopherol (or α-tocopherol/astaxanthin: 0.6) as the optimum concentrations which would give the maximum stability to astaxanthin once added to produced nanodispersion systems. Furthermore, in order to ensure the physical stability of all studied nanodispersions, future studies may also focus on the production of pH stable nanodispersion systems, for example, by the optimization of buffer concentration.
